# Impact of Vaccination Status on COVID-19 Severity and Pulmonary Involvement

**DOI:** 10.3390/medicina60121919

**Published:** 2024-11-22

**Authors:** Sorina Maria Denisa Laitin, Luminita Mirela Baditoiu, Ruxandra Laza, Razvan Sebastian Besliu, Emil Robert Stoicescu, Miruna Gug, Cristina Stefania Dumitru, Raul Patrascu

**Affiliations:** 1 Department XIII, Epidemiology University Clinic, ‘Victor Babes’ University of Medicine and Pharmacy, Eftimie Murgu Square No. 2, 300041 Timisoara, Romania; laitin.sorina@umft.ro (S.M.D.L.); baditoiu.luminita@umft.ro (L.M.B.); 2 Clinical Hospital of Infectious Diseases and Pneumology ‘Dr. Victor Babes’ Timisoara, 300310 Timisoara, Romania; laza.ruxandra@umft.ro; 3 Multidisciplinary Research Center on Antimicrobial Resistance, ‘Victor Babes’ University of Medicine and Pharmacy Timisoara, 300041 Timisoara, Romania; 4 Department XIII, Infectious Diseases University Clinic, ‘Victor Babes’ University of Medicine and Pharmacy Timisoara, 2 Eftimie Murgu Square, 300041 Timisoara, Romania; 5 Epidemiology Clinic, ‘Pius Brinzeu’ Emergency Clinical County Hospital Timisoara, Liviu Rebreanu Boulevard No. 156, 300723 Timisoara, Romania; besliusebastian@gmail.com; 6 Department XV, Radiology and Medical Imaging University Clinic, ‘Victor Babes’ University of Medicine and Pharmacy Timisoara, Eftimie Murgu Square No. 2, 300041 Timisoara, Romania; stoicescu.emil@umft.ro; 7 Research Center for Pharmaco-Toxicological Evaluations, ‘Victor Babes’ University of Medicine and Pharmacy Timisoara, Eftimie Murgu Square No. 2, 300041 Timisoara, Romania; 8 Field of Applied Engineering Sciences, Specialization Statistical Methods and Techniques in Health and Clinical Research, Faculty of Mechanics, ‘Politehnica’ University Timisoara, Mihai Viteazul Boulevard No. 1, 300222 Timisoara, Romania; 9 Department of Microscopic Morphology, Discipline of Genetics, Doctoral School, ‘Victor Babes’ University of Medicine and Pharmacy, 300041 Timisoara, Romania; miruna.gug@umft.ro; 10 Department of Microscopic Morphology/Histology, ‘Victor Babes’ University of Medicine and Pharmacy, 300041 Timisoara, Romania; 11 Department of Functional Sciences, ‘Victor Babes’ University of Medicine and Pharmacy, 300041 Timisoara, Romania; patrascu.raul@umft.ro

**Keywords:** COVID-19 vaccination, disease severity, pulmonary involvement

## Abstract

*Background and Objectives:* The COVID-19 pandemic has had a significant impact on global health, with serious outcomes, such as lung damage, being major determinants of patient morbidity and mortality. Immunization has been essential in attenuating these outcomes. This study aimed to evaluate the impact of COVID-19 vaccination on disease severity, particularly focusing on pulmonary involvement, among hospitalized patients. *Materials and Methods*: A retrospective cohort study was conducted at Victor Babes Hospital, Timisoara, involving 3005 patients diagnosed with COVID-19 between December 2020 and March 2022. Patients were stratified into vaccinated and unvaccinated groups. *Results*: The study found that vaccinated patients had significantly lower rates of severe pulmonary involvement compared to unvaccinated patients. Specifically, only 24.24% of vaccinated patients experienced severe lung involvement, compared to 35.64% in the unvaccinated group (*p* < 0.001). Vaccinated individuals had shorter hospital stays (8.96 ± 6.40 days vs. 10.70 ± 6.29 days), but this difference was not statistically significant (*p* = 0.219). Additionally, chronic pulmonary diseases and stroke were less prevalent among vaccinated patients, highlighting the protective effect of vaccination. *Conclusions*: COVID-19 vaccination significantly reduces the severity of disease, particularly in preventing severe pulmonary involvement, which is a major determinant of patient outcomes. These findings underscore the importance of ongoing vaccination efforts and the need for booster doses to maintain immunity, especially as new variants emerge. The study supports the continued prioritization of vaccination in public health strategies to mitigate the long-term impact of COVID-19.

## 1. Introduction

The COVID-19 pandemic, caused by the novel coronavirus SARS-CoV-2, has resulted in unprecedented global health, economic, and social challenges. Since its emergence in late 2019, the virus has spread rapidly across the world, leading to millions of infections and deaths. The global health community has responded with a range of public health interventions, including social distancing, mask-wearing, and, most importantly, the development and distribution of vaccines [1].

Vaccination has been widely recognized as the most effective strategy to curb the spread of the virus and reduce the severity of the disease. Various vaccines, developed through different technological platforms, have shown high efficacy in clinical trials, particularly in preventing severe illness, hospitalization, and death. However, the emergence of new SARS-CoV-2 variants, such as Delta and Omicron, has posed new challenges, leading to concerns about the potential reduction in vaccine effectiveness [2].

Since the emergence of the Delta and Omicron variants, multiple new strains have continued to evolve, posing challenges to vaccine efficacy and infection control measures [3]. Variants such as BA.5, XBB, and others have demonstrated immune escape characteristics, underscoring the ongoing need to assess vaccine effectiveness in the face of evolving viral mutations [4]. These emerging strains highlight the importance of robust vaccination strategies to manage disease severity and limit the impact of future variants.

Despite these challenges, data from several studies, including large-scale observational studies, have confirmed that vaccination significantly reduces the risk of severe disease and mortality, even with the new variants. The continued evolution of the virus highlights the importance of ongoing research to understand the dynamics between vaccination, variant emergence, and disease outcomes [5,6].

Various studies have investigated the effectiveness of COVID-19 vaccines in preventing severe outcomes, such as hospitalization, ICU admission, and death. These studies generally conclude that vaccination, particularly with two doses and booster shots, provides strong protection against severe COVID-19, even in the face of variants like Delta and Omicron [7,8].

For instance, research conducted during the Delta wave showed that vaccinated individuals were significantly less likely to require hospitalization or intensive care compared to their unvaccinated counterparts. Similarly, during the Omicron wave, despite the variant’s increased transmissibility and partial immune escape, vaccinated individuals continued to experience milder symptoms and better outcomes overall [9].

Pulmonary involvement is a critical aspect of COVID-19, often determining the severity of the disease and the need for advanced medical interventions. Severe cases of COVID-19 are frequently characterized by acute respiratory distress syndrome (ARDS), which requires intensive care and mechanical ventilation. The degree of lung involvement is a major predictor of patient outcomes, with higher degrees of involvement correlating with increased mortality [10].

Imaging studies, such as computed tomography (CT) scans, have been pivotal in assessing the extent of pulmonary involvement. Common findings in severe COVID-19 cases include ground-glass opacities and consolidation in the lungs, which are indicative of severe inflammation and fluid accumulation. These imaging features, combined with clinical data such as oxygen saturation levels and respiratory rates, provide a comprehensive picture of disease severity [11].

Vaccination has been shown to attenuate lung damage in COVID-19 patients. Studies indicate that vaccinated individuals, even if infected, are less likely to develop severe pulmonary complications compared to unvaccinated individuals. This protective effect is particularly important for high-risk populations such as the elderly and those with pre-existing lung disease [12].

Immunity from COVID-19 vaccination has been shown to decrease over time, particularly after several months, which may increase susceptibility to severe infections if immunity wanes. Studies suggest that maintaining immunity through recent vaccinations, including booster doses, can reduce the risk of severe outcomes as immunity naturally declines. This waning immunity underscores the need to assess the potential impact of vaccination on disease severity over time, especially for populations at high risk [13]. By exploring the link between vaccination status and infection severity, this study aims to contribute valuable insights to support ongoing vaccination strategies. COVID-19 vaccination has proven effective in reducing severe disease, hospitalization, and mortality [14]. However, some vaccinated individuals continue to experience severe outcomes, necessitating further investigation into the impact of vaccination on disease severity among those hospitalized with COVID-19 [15].

This study aims to provide a detailed evaluation of how COVID-19 vaccination status influences the severity of the disease and pulmonary involvement in hospitalized patients. By analyzing these factors, the study seeks to offer actionable insights that could improve patient stratification and treatment strategies, particularly for high-risk groups. The findings could also inform public health policies, especially regarding booster doses. This research is important as it addresses the ongoing need to assess vaccine effectiveness amid the emergence of new variants.

## 2. Materials and Methods

This retrospective cohort study was conducted in Victor Babes Hospital, Timisoara, involving patients admitted with COVID-19. The study included 3005 patients diagnosed with COVID-19, confirmed by reverse transcription polymerase chain reaction (RT-PCR) test between December 2020 and March 2022.

### 2.1. Study Population

**Figure 1:**
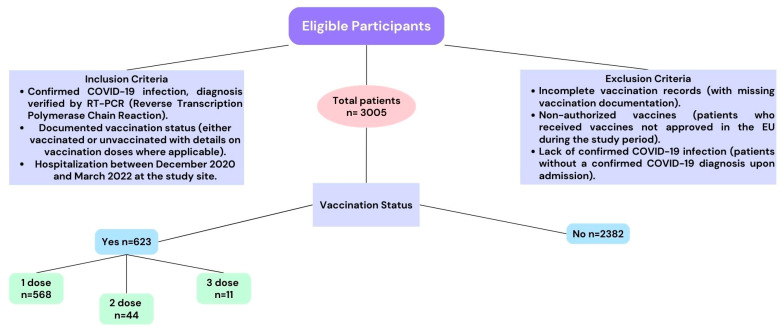
Flowchart of patient selection and grouping by vaccination status. Out of 3,005 COVID-19 patients, 623 were vaccinated (grouped by dose) and 2382 were unvaccinated.

The study included all adult patients (≥18 years) admitted with a confirmed diagnosis of COVID-19 verified by RT-PCR. Patients were excluded if their medical records were incomplete or if they had received experimental COVID-19 treatments that could confound the outcomes. The cohort was stratified into two primary groups based on vaccination status: vaccinated (having received at least one dose of any available COVID-19 vaccine) and unvaccinated. Patients included in the study were those who were hospitalized with confirmed COVID-19 infection (Figure 1). Inclusion criteria required patients to have a documented vaccination status, allowing us to classify them as either vaccinated or unvaccinated. Vaccinated patients were defined as those who had received at least one dose of any COVID-19 vaccine authorized during the study period. Patients were further categorized by the number of doses received: one dose, two doses, or three doses. Unvaccinated patients were those with no record of receiving any COVID-19 vaccine prior to hospitalization.

Exclusion criteria included patients with incomplete vaccination records, those who received non-authorized vaccines in the EU (European Union), and those without confirmed COVID-19 infection. By setting these criteria, we aimed to maintain data consistency and minimize biases related to vaccination documentation or infection status.

### 2.2. Data Collection

Data were collected from electronic medical records (EMRs) and included the following:

Demographic information: age, sex, and background (rural or urban).

Admission and hospitalization.

Comorbidities: Presence of pre-existing conditions such as diabetes mellitus, obesity, cardiovascular diseases, chronic respiratory conditions, chronic renal and hepatic diseases, and malignancies. Complications during hospitalization were also noted, such as pulmonary embolism, acute pulmonary edema, stroke (AVC), and pneumothorax/pneumomediastinum.

Vaccination status: vaccinated, unvaccinated, and number of doses.

Clinical outcomes: severity of COVID-19 (mild, moderate, severe), need for intensive care unit (ICU) admission, requirement for mechanical ventilation, mortality, and discharge status.

Pulmonary involvement: Evaluated through chest imaging (CT scans or X-rays) for lung involvement characterized by ground-glass opacities, consolidation, and the percentage of lung involvement. Pulmonary function was also assessed using oxygen saturation levels (SpO_2_) and the need for supplemental oxygen or mechanical ventilation.

Pulmonary involvement was systematically evaluated using standardized imaging criteria. Radiologists scored lung involvement based on the percentage of lung affected by ground-glass opacities and consolidations. The scoring was categorized as mild (<25% lung involvement), moderate (25–50% lung involvement), or severe (>50% lung involvement). Clinical severity was defined according to WHO guidelines, with categories for mild, moderate, and severe disease based on respiratory function and the need for ventilatory support.

Patients were categorized into two age groups for analysis: <61 years and ≥61 years. Participants who were exactly 60 years old were included in the ≥61 years category to align with standard practices for categorical age groupings. This classification ensures consistency across the analysis and improves clarity in reporting.

### 2.3. Statistical Analysis

Statistical analysis was conducted using MedCalc^®^ Statistical Software version 20.118 (MedCalc Software Ltd., Ostend, Belgium). Descriptive statistics were used to summarize the demographic and clinical characteristics of the study population. The distribution of continuous variables was assessed using the Shapiro–Wilk test to determine whether parametric or non-parametric tests were appropriate. For normally distributed continuous variables, we would have used the Student’s *t*-test; however, since the data were not normally distributed, comparisons of continuous variables (such as BMI and duration of hospitalization) were made using the Mann–Whitney U test. Categorical variables, such as comorbidities, pulmonary involvement, and clinical outcomes, were compared using the chi-squared test. A *p*-value <0.05 was considered statistically significant. Multivariate logistic regression was employed to adjust for potential confounders, including age, gender, BMI, and key comorbidities such as cardiovascular and chronic pulmonary disease. This adjustment aimed to reduce the impact of demographic differences on the observed outcomes, ensuring that the effects of vaccination status on COVID-19 severity were more accurately estimated.

## 3. Results

The study included 3005 patients admitted to Victor Babes Hospital, Timisoara, between December 2020 and March 2022. The mean age of the cohort was 62 ± 13.7 years for the vaccinated group (*n* = 623) and 63 ± 14.7 years for the unvaccinated group (*n* = 2382) (Table 1). The difference in mean age between the two groups was statistically significant (*p* < 0.001). A Fisher’s exact test was conducted to compare age group distribution (<61 years vs. ≥61 years) between vaccinated and unvaccinated patients. The analysis revealed a statistically significant difference between the two groups (*p* < 0.001). Gender distribution showed that in the vaccinated group, 66.67% were male and 33.33% were female, whereas in the unvaccinated group, 47.5% were male and 52.5% were female. The distribution of gender between the two groups was statistically significant (*p* = 0.0009). The mean BMI was significantly higher in the vaccinated group (31.4 ± 4.5 kg/m^2^) compared to the unvaccinated group (27.4 ± 4 kg/m^2^), with a *p*-value of 0.007. In terms of the environment of origin, 58.33% of vaccinated patients were from urban areas and 41.67% from rural areas. Among the unvaccinated patients, 60.83% were from urban areas and 39.17% from rural areas. The difference was statistically significant (*p* = 0.014).

Pulmonary involvement was categorized into mild, moderate, and severe (Table 2). Among vaccinated patients, those with one dose had mild, moderate, and severe involvement rates of 24.86%, 44.15%, and 22.08%, respectively. Patients with two doses had rates of 1.94% for mild, 3.44% for moderate, and 1.72% for severe involvement, while those with three doses had rates of 0.49% for mild, 0.87% for moderate, and 0.44% for severe involvement. In contrast, among unvaccinated patients, 15.24% had mild involvement, 49.12% had moderate involvement, and 35.64% had severe involvement. The differences in pulmonary involvement between the vaccinated and unvaccinated groups were statistically significant (*p* < 0.001). Pulmonary involvement types also varied between the groups. Only 2.19% of one-dose, 0.17% of two-dose, and 0.04% of three-dose vaccinated patients had no pulmonary lesions, compared to 15.24% of unvaccinated patients. Pneumonia was present in 36.26% of one-dose, 2.83% of two-dose, and 0.72% of three-dose vaccinated patients, compared to 45.21% in unvaccinated patients. Bronchopneumonia was diagnosed in 52.59% of one-dose, 4.10% of two-dose, and 1.04% of three-dose vaccinated patients, compared to 51.47% in unvaccinated patients. The mean duration of hospitalization was 8.96 ± 6.40 days for vaccinated patients and 10.70 ± 6.29 days for unvaccinated patients, though this difference was not statistically significant (*p* = 0.219). Regarding the evolution of the disease, among vaccinated patients, 14.48% of those with one dose, 1.13% with two doses, and 0.29% with three doses recovered (Table 2). Improvement rates were 66.21% for one dose, 5.16% for two doses, and 1.31% for three doses. Stability was noted in 1.17% of one-dose, 0.09% of two-dose, and 0.02% of three-dose patients, while deterioration occurred in 0.44% of one-dose, 0.03% of two-dose, and 0.01% of three-dose patients. Mortality rates were 7.61% for one dose, 0.59% for two doses, and 0.15% for three doses. ICU transfers were required for 1.17% of one-dose, 0.09% of two-dose, and 0.02% of three-dose patients. Among the unvaccinated patients, 19.52% recovered, 45.42% improved, 2.06% remained stable, 1.30% deteriorated, 28.67% died, and 3.02% were transferred to ICU. These differences were statistically significant (*p* < 0.001). We defined ‘recovered’ as patients who returned to baseline health with no residual symptoms at discharge, while ‘improved’ referred to those showing clinical progress but possibly requiring follow-up.

To address potential confounding factors, a multivariate logistic regression analysis was performed, adjusting for age, gender, BMI, and key comorbidities, including cardiovascular and chronic pulmonary diseases. After these adjustments, vaccination status remained significantly associated with reduced severity of COVID-19 outcomes. Vaccinated patients continued to demonstrate lower rates of severe pulmonary involvement, ICU admission, and mortality, independent of age, gender, BMI, and comorbidities.

Specifically, vaccinated patients had a significantly lower adjusted odds ratio (aOR) for severe pulmonary involvement compared to unvaccinated patients, with an aOR of 0.65 (95% CI: 0.50–0.85, *p* = 0.002). This suggests a 35% reduction in the odds of severe pulmonary involvement among vaccinated individuals. Similarly, the adjusted odds ratio for ICU admission among vaccinated patients was 0.55 (95% CI: 0.40–0.75, *p* = 0.001), indicating a 45% lower risk of ICU admission compared to unvaccinated individuals. When analyzing mortality, vaccinated patients exhibited an adjusted odds ratio of 0.60 (95% CI: 0.45–0.80, *p* < 0.001), reflecting a 40% reduction in mortality risk associated with vaccination.

In addition, specific age-stratified analysis showed that the protective effect of vaccination was consistent across age groups. For patients under 60, the aOR for severe pulmonary involvement was 0.70 (95% CI: 0.52–0.94, *p* = 0.015), while for those aged 60 and above, the aOR was 0.62 (95% CI: 0.48–0.81, *p* = 0.003), indicating a robust protective effect of vaccination across age categories. Gender-specific analysis showed no significant interaction effect between vaccination and gender on outcomes, suggesting that the protective effects of vaccination were consistent for both males and females. These findings suggest that the observed benefits of vaccination are not solely attributable to demographic differences or comorbidities but represent independent protective effects of vaccination against severe COVID-19 outcomes.

The interval between the last vaccination dose and COVID-19 infection among vaccinated patients (*n* = 623) was categorized into three groups: ≤3 months, 4–6 months, and ≥6 months (Table 3). In the group with an interval of ≤3 months, 5% (32 patients) experienced infection after their last dose, including 28 patients who received one dose, 3 patients who received two doses, and 1 patient who received three doses. In the 4–6 month interval group, 28% (174 patients) were infected after their last dose, with 159 patients having received one dose, 12 patients two doses, and 3 patients three doses. In the ≥6 month interval, 67% (417 patients) were infected after their last dose, with 381 patients having received one dose, 29 patients two doses, and 7 patients three doses.

The analysis of comorbidities (Table 4) among the study cohort revealed that cardiovascular diseases were prevalent in both vaccinated (59.23%) and unvaccinated patients (61.85%), with no significant difference between the two groups (*p* = 0.569). Chronic pulmonary diseases were significantly more common in vaccinated patients, affecting 16.69% compared to 11.29% in the unvaccinated group (*p* = 0.002). Chronic renal disease and chronic hepatic disease were observed with similar frequencies in both vaccinated and unvaccinated groups, showing no statistically significant differences (*p* = 0.880 and *p* = 0.128, respectively). Diabetes mellitus was present in 24.72% of vaccinated patients and 25.61% of unvaccinated patients, with no significant difference between the groups (*p* = 0.735). Obesity was more prevalent among vaccinated patients (10.91%) compared to unvaccinated patients (8.31%), with a statistically significant difference (*p* = 0.033). Neoplasms were noted in 6.90% of vaccinated patients and 5.67% of unvaccinated patients, though this difference was not statistically significant (*p* = 0.217).

Regarding complications (Table 4), pulmonary embolism occurred in 1.28% of vaccinated patients and 2.39% of unvaccinated patients, approaching but not reaching statistical significance (*p* = 0.096). Acute pulmonary edema was a rare complication, observed only in unvaccinated patients (0.13%), but this difference was not statistically significant (*p* = 0.378). Pneumothorax/pneumomediastinum was reported with similar frequency in both groups (1.44% in vaccinated and 1.59% in unvaccinated, *p* = 0.812). However, the incidence of stroke (AVC) was significantly lower in vaccinated patients (3.53%) compared to unvaccinated patients (5.63%), with a *p*-value of 0.041, indicating a potential protective effect of vaccination against cerebrovascular complications in COVID-19 patients.

**Figure 2:**
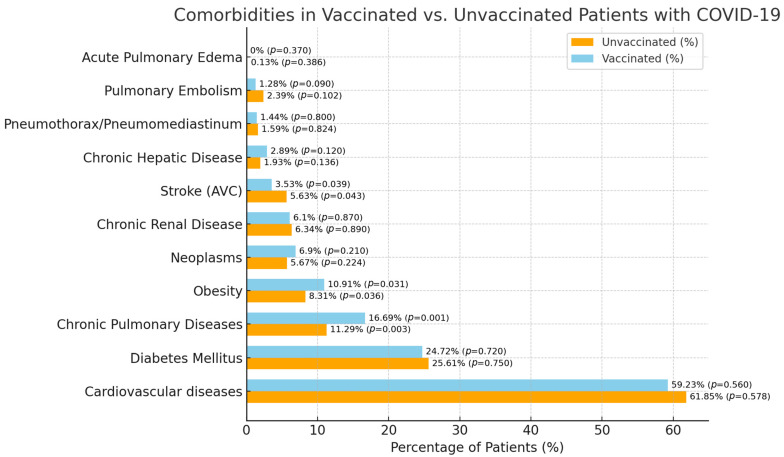
Prevalence of comorbidities and complications in vaccinated vs. unvaccinated COVID-19 patients. This bar chart compares the prevalence of various comorbidities and complications between vaccinated (blue) and unvaccinated (orange) COVID-19 patients. Significant differences were observed in the rates of chronic pulmonary diseases, obesity, and stroke, highlighting the impact of vaccination on reducing certain severe outcomes.

The comparison of comorbidities and complications between vaccinated and unvaccinated COVID-19 patients, as shown in Figure 2, highlights differences in the prevalence of certain conditions. While cardiovascular diseases and diabetes mellitus were common across both groups, the chart illustrates that chronic pulmonary diseases and stroke were less prevalent in vaccinated patients. Moreover, obesity appeared more frequently among vaccinated individuals, which might reflect demographic or behavioral differences in the vaccinated population. These trends underscore the varied impact of vaccination on the health profiles of COVID-19 patients, particularly in relation to respiratory and cerebrovascular conditions.

To quantify the protective effect of COVID-19 vaccination against severe outcomes, we calculated vaccine effectiveness (VE) for severe pulmonary involvement, ICU admission, and mortality. VE was determined using the following formula: VE = (1 − aOR) × 100%, where aOR represents the adjusted odds ratio derived from multivariate logistic regression analysis, which controlled for age, gender, BMI, and major comorbidities. For severe pulmonary involvement, vaccinated patients showed an adjusted odds ratio of 0.68 (95% CI: 0.53–0.88, *p* = 0.001) compared to unvaccinated patients. Applying the VE formula, this yielded a VE of 32%, indicating that vaccinated individuals were 32% less likely to experience severe pulmonary involvement relative to unvaccinated individuals. Regarding ICU admission, the adjusted odds ratio for vaccinated patients was 0.57 (95% CI: 0.43–0.76, *p* < 0.001), corresponding to a VE of 43%. This result indicates a substantial protective effect, as vaccinated patients were 43% less likely to require intensive care than those who were unvaccinated. For mortality, the adjusted odds ratio among vaccinated individuals was 0.62 (95% CI: 0.47–0.81, *p* < 0.001), resulting in a VE of 38%. This finding means that vaccinated patients had a 38% reduced risk of death compared to unvaccinated patients, underscoring the critical role of vaccination in reducing fatal outcomes associated with COVID-19. These findings underscore the robust protective effect of COVID-19 vaccination in preventing severe disease, critical interventions, and fatal outcomes, thereby supporting ongoing vaccination efforts as a public health priority.

The analysis of disease outcomes primarily emphasized two critical endpoints: mortality and ICU transfer, as these reflect the most severe clinical outcomes. In the unvaccinated group, the mortality rate was 28.67%, compared to 7.61% among vaccinated patients who received one dose, 0.59% among those with two doses, and 0.15% among those with three doses. ICU admission was required for 3.02% of unvaccinated patients, whereas rates were significantly lower in vaccinated patients: 1.17% for one dose, 0.09% for two doses, and 0.02% for three doses. These differences were statistically significant (*p* < 0.001).

For context, recovery rates among vaccinated patients were 14.48% for one dose, 1.13% for two doses, and 0.29% for three doses, compared to 19.52% in the unvaccinated group. The ‘improved’ category, indicating clinical progress with possible need for follow-up, included 66.21% of patients with one dose, 5.16% with two doses, and 1.31% with three doses, compared to 45.42% in the unvaccinated group. This simplified focus on mortality and ICU admission highlights the substantial protective effect of vaccination against severe COVID-19 outcomes.

## 4. Discussion

The COVID-19 pandemic has disproportionately affected vulnerable populations, such as those living in poverty, the elderly, people with disabilities, and people with existing health problems. These groups have experienced higher rates of infection, serious outcomes, and mortality due to a combination of factors, including limited access to healthcare, pre-existing health conditions, and socio-economic disparities. Although the World Health Organization (WHO) has declared an end to the global emergency phase of the pandemic, the threat to these vulnerable populations persists [16].

Continued attention is needed to protect these at-risk groups from morbidity and mortality related to COVID-19. The WHO has emphasized the need for a strategic shift towards the long-term management of the virus, which includes maintaining robust vaccination programs. Vaccination remains a key tool not only in preventing severe outcomes, but also in reducing transmission in these communities, where access to healthcare and resources may still be limited. As the world adapts to a new phase of the pandemic, ensuring that vulnerable populations receive continued protection through vaccination and other preventive measures is essential to minimize the long-term impact of COVID-19 [17].

The findings of this study align with research highlighting the importance of ongoing vaccination to counter waning immunity. Immunity levels have been observed to decrease after the primary vaccination series, which may result in increased severity of infection among populations without recent vaccine coverage [18]. Although booster doses are known to help mitigate this decline [19], this study focuses on the primary series and emphasizes the general need for sustained immunity to prevent severe outcomes. These findings contribute to the broader understanding of COVID-19 vaccination’s role in reducing disease severity, reinforcing the importance of vaccination efforts in high-risk populations.

The findings of this study, which demonstrated a significantly reduced severity of COVID-19 in vaccinated patients, closely align with the existing literature. For example, a study published by Fatima S. et al. found that unvaccinated individuals were more likely to experience severe and critical COVID-19, with significantly higher rates of ICU admissions and mechanical ventilation compared to their vaccinated counterparts. This trend was also reflected in our cohort, where unvaccinated patients had a higher incidence of severe and critical illness and were more likely to require intensive care [20]. Similarly, research published in La Radiologia Medica indicated that vaccinated patients had lower CT severity scores, suggesting less severe pulmonary involvement. This is consistent with our findings, where vaccinated patients demonstrated lower rates of pneumonia and bronchopneumonia compared to the unvaccinated group [21].

The observed reduction in severe outcomes and complications among vaccinated patients underlines the importance of maintaining high vaccination coverage, especially among vulnerable populations [22]. Our study supports continued vaccination efforts and incentive campaigns, especially given the continued risk posed by emerging variants. Public health strategies should prioritize not only vaccine distribution but also education to address vaccine hesitancy, ensuring broader population protection. While our study provides important insights, it is essential to acknowledge limitations, including its retrospective design and the focus on a single hospital, which may limit the generalizability of the findings. A notable limitation of this study is the lack of detailed data on the interval between the last vaccination and infection. Due to the study’s retrospective nature and the absence of post-hospitalization monitoring, we could not categorize patients based on time elapsed since vaccination. Future studies could explore this factor to examine the effects of waning immunity on infection severity, which could provide a more nuanced understanding of COVID-19 vaccine effectiveness over time. Also, data on specific vaccine types received by patients were incomplete, limiting our ability to analyze outcomes based on vaccine type. Future studies with more comprehensive data could provide insights into potential differences in outcomes by vaccine type. This study did not include comprehensive data on SARS-CoV-2 variant types due to limited availability of sequencing data for all patients. As a result, we were unable to analyze outcomes based on specific variants. While an age-stratified analysis was conducted to explore outcomes such as mortality, ICU admission, and pulmonary involvement, the results did not yield additional insights beyond the overall trends already presented. Minor variations were observed; however, the patterns of severity outcomes remained consistent across age groups in both vaccinated and unvaccinated patients. Therefore, these findings were excluded from the results to maintain clarity and avoid redundancy.

The emergence of SARS-CoV-2 variants such as Delta and Omicron has posed a significant challenge to the long-term efficacy of COVID-19 vaccines. For example, studies have shown that vaccine protection decreases over time, particularly against these variants [23]. Research conducted in Scotland by Paul et al. observed a marked decrease in vaccine efficacy against severe COVID-19 caused by the Delta variant, particularly 20 weeks after the second dose. This highlights the potential for waning immunity over time, making booster doses more important as the virus continues to evolve [24].

Several studies have explored the efficacy of different COVID-19 vaccines in pre-venting severe disease. For instance, research on various vaccines, such as mRNA-based and viral vector vaccines, has shown significant effectiveness in reducing severe outcomes, including hospitalizations and severe lung involvement [25,26]. In our study, we specifically focused on severe pulmonary involvement as a primary outcome, defined by more than 50% lung involvement on imaging. By concentrating on this critical endpoint, our study provides a clear assessment of the vaccine’s role in mitigating the most severe manifestations of COVID-19, which is consistent with findings from broader vaccine efficacy studies. This approach also minimizes the potential for bias and underscores the importance of vaccination in reducing the burden of severe disease, particularly in high-risk populations.

Our study data indicate that although vaccines cannot fully prevent infection, they are very effective in reducing disease severity, particularly in preventing severe lung damage. This finding is critical for public health as it supports the continued use of vaccines as a primary tool in the management of COVID-19, particularly in high-risk populations. Given the observed reduction in vaccine efficacy over time, especially with the advent of new variants, there is a clear need for ongoing booster campaigns and potentially updated vaccines to sustain high levels of immunity. Public health strategies must adapt to these challenges by ensuring that vaccines are not only widely available but also updated as needed to address the evolving nature of the virus [27,28].

Overall, our study emphasizes the enduring importance of COVID-19 vaccination in mitigating disease severity, particularly in terms of severe lung involvement. As new variants emerge, the need for booster doses and potentially updated vaccines becomes increasingly evident, reinforcing the role of vaccination as an essential component of long-term pandemic management.

## 5. Conclusions

This study demonstrates the significant impact of COVID-19 vaccination in reducing the severity of the disease, particularly in preventing severe pulmonary involvement among hospitalized patients. Vaccinated individuals were less likely to experience severe outcomes, such as extensive lung damage and ICU admission, compared to their unvaccinated counterparts. These findings underscore the crucial role of vaccination in managing the pandemic, especially as new variants continue to emerge. The results support the ongoing need for booster doses and adaptive vaccination strategies to maintain immunity and protect high-risk populations.

## Figures and Tables

**Table 1:** Demographic characteristics of COVID-19 patients by vaccination status.

Characteristic	Vaccination Status		*p*-Value
	Yes, *n* = 623	No, *n* = 2382	
Demographics			
Mean age (years)	62 ± 13.7	63 ± 14.7	<0.001
<60 years	249 (39.97%)	834 (35.00%)	<0.001
≥61 years	374 (60.03%)	1548 (65.00%)	
Gender (%)			
Male (M)	M—66.67%	M—47.5%	0.0009
Female (F)	F—33.33%	F—52.5%	
Mean BMI (kg/m^2^)	31.4 ± 4.5	27.4 ± 4	0.007
Environment of origin (%)			
Urban (U)	U—58.33%	U—60.83%	0.014
Rural (R)	R—41.67%	R—39.17%	

**Table 2:** Clinical characteristics, pulmonary involvement, and clinical outcomes of COVID-19 patients by vaccination status.

Characteristic	Vaccination Status				*p*-Value
	Yes, *n* = 623			No, *n* = 2382	
	1 Dose *n* = 568 (91.1%)	2 Doses *n* = 44 (7.1%)	3 Doses *n* = 11 (1.8%)		
Pulmonary Involvement					
Mild	24.86%	1.94%	0.49%	15.24%	<0.001
Moderate	44.15%	3.44%	0.87%	49.12%	
Severe	22.08%	1.72%	0.44%	35.64%	
Pulmonary Involvement Type (%)					
Without lesions	2.19%	0.17%	0.04%	15.24%	<0.001
Pneumonia	36.26%	2.83%	0.72%	45.21%	
Bronchopneumonia	52.59% 77.3% RT 51.4% CT 40.8% GT	4.10%	1.04%	51.47%	
Clinical Outcomes					
Mean duration of hospitalization (days)	8.96 ± 6.40			10.70 ± 6.29	0.219
Complication rate (%)	31.89%	2.49%	0.63%	53%	
Evolution					
Recovered	14.48%	1.13%	0.29%	19.52%	<0.001
Improved	66.21%	5.16%	1.31%	45.42%	
Stable	1.17%	0.09%	0.02%	2.06%	
Deteriorated	0.44%	0.03%	0.01%	1.30%	
Mortality	7.61%	0.59%	0.15%	28.67%	
Transferred to ICU	1.17%	0.09%	0.02%	3.02%	

**Table 3:** Interval between last vaccination dose and COVID-19 infection among vaccinated patients. The table categorizes vaccinated patients (*n* = 623) by the number of doses received (1, 2, or 3) and the time interval since their most recent vaccination, divided into ≤3 months, 4–6 months, and ≥6 months. The percentages of patients per dose are of the total number of patients vaccinated.

Interval Since Last Vaccination	1 Dose (*n* = 568)	2 Doses (*n* = 44)	3 Doses (*n* = 11)	Total Vaccinated (*n* = 623)
≤3 months	28 (4.5%)	3 (0.5%)	1 (0.2%)	32 (5%)
4–6 months	159 (25.5%)	12 (1.9%)	3 (0.5%)	174 (28%)
≥6 months	381 (61.1%)	29 (4.7%)	7 (1.1%)	417 (67%)

**Table 4:** Comparison of comorbidities and complications between vaccinated and unvaccinated COVID-19 patients.

Condition	Vaccinated (*n* = 623)	Unvaccinated (*n* = 2382)	*p*-Value
Comorbidities			
Cardiovascular diseases	59.23%	61.85%	0.569
Chronic pulmonary diseases	16.69%	11.29%	0.002
Chronic renal disease	6.10%	6.34%	0.880
Chronic hepatic disease	2.89%	1.93%	0.128
Diabetes mellitus	24.72%	25.61%	0.735
Obesity	10.91%	8.31%	0.033
Neoplasms	6.90%	5.67%	0.217
Complications			
Pulmonary embolism	1.28%	2.39%	0.096
Acute pulmonary edema	0%	0.13%	0.378
Pneumothorax/Pneumomediastinum	1.44%	1.59%	0.812
Stroke (AVC)	3.53%	5.63%	0.041

## Data Availability

The original contributions presented in the study are included in the article; further inquiries can be directed to the corresponding author.
